# The isotype and IgG subclass distribution of anti-carbamylated protein antibodies in rheumatoid arthritis patients

**DOI:** 10.1186/s13075-017-1392-z

**Published:** 2017-08-15

**Authors:** Myrthe A. M. van Delft, Marije K. Verheul, Leonie E. Burgers, Veerle F. A. M. Derksen, Annette H. M. van der Helm-van Mil, Diane van der Woude, Tom W. J. Huizinga, René E. M. Toes, Leendert A. Trouw

**Affiliations:** Department of Rheumatology, C1-R, Leiden University Medical Center, PO Box 9600, 2300 RC Leiden, The Netherlands

**Keywords:** Autoantibodies, anti-CarP antibodies, ACPA, Rheumatoid arthritis, Antibody isotypes, Antibody IgG subclasses

## Abstract

**Background:**

Anti-carbamylated protein (anti-CarP) antibodies have recently been reported to occur in around 45% of rheumatoid arthritis (RA) patients and to have prognostic and diagnostic properties. At present, the breadth and molecular make-up of the anti-CarP antibody response is ill defined. To understand the anti-CarP antibody immune response and potential immune effector mechanisms it can recruit, we determined the anti-CarP antibody isotype and IgG-subclass usage in RA patients.

**Methods:**

Anti-CarP antibody IgM, IgA, and IgG or IgG subclasses were detected by enzyme-linked immunosorbent assay (ELISA) in sera from 373 unselected RA patients and 196 healthy controls. An additional 114 anti-citrullinated protein antibody (ACPA) and anti-CarP IgG double-positive patients were selected to study the concomitant presence of both antibody systems.

**Results:**

Anti-CarP IgG was present in around 45% of the patients and comprised all anti-CarP IgG subclasses. The presence of anti-CarP IgG1 particularly associates with radiological damage. Anti-CarP IgM was detected in 16% of RA patients, even in anti-CarP IgG-positive individuals, and is indicative of an actively ongoing immune response. Around 45% of the patients were positive for IgA which included ACPA-positive cases but also 24% of the ACPA-negative cases. In ACPA and anti-CarP double-positive patients, the distribution and number of isotypes and IgG subclasses was similar for both autoantibodies at the group level, but substantial variation was observed within individual patient samples.

**Conclusions:**

In RA, the anti-CarP antibody response uses a broad spectrum of isotypes and seems to be an actively ongoing immune reaction. Furthermore, the anti-CarP and ACPA autoantibody responses seems to be differentially regulated.

**Electronic supplementary material:**

The online version of this article (doi:10.1186/s13075-017-1392-z) contains supplementary material, which is available to authorized users.

## Background

Rheumatoid arthritis (RA) is a systemic autoimmune disease principally affecting synovial joints [1, 2]. Autoantibodies can be detected in the serum and synovial fluid (SF) of RA patients and may form immune complexes in the joints, leading to the attraction of immune cells through, for example, complement activation [3, 4] which can contribute to chronic inflammation and bone destruction.

Several autoantibodies have been described in RA. Well-known biomarkers that are currently used in the clinic for the diagnosis of RA are rheumatoid factor (RF) and anti-citrullinated protein antibodies (ACPAs) [5]. More recently, anti-carbamylated protein (anti-CarP) antibodies, which target carbamylated proteins, have been detected in RA [6]. Carbamylation is a post-translational modification of proteins in which lysines are converted to homocitrullines by a chemical reaction with cyanate [7, 8].

Currently, several studies have shown an increase in the prevalence of anti-CarP antibodies in RA patients [6, 9–11] and, like ACPA, the presence of anti-CarP antibodies associates with increased joint destruction [6, 9, 10, 12]. Moreover, anti-CarP antibodies are already present in individuals before disease onset [9, 13, 14] and the presence of these antibodies is predictive for the progression to RA in arthralgia patients [15]. Overall, these data indicate that anti-CarP antibodies might play a role in the disease pathogenesis of RA, although little is known about the precise constitution of the anti-CarP antibody response or how the make-up of this response may or may not play a role in disease development.

In humans, several immunoglobulin (Ig) isotypes are known. During a primary immune response (first antigen encounter) activated B cells will secrete IgM. During the subsequent maturation of an immune response, isotype switching occurs which leads to an augmented diversity of the antibody response. In case of T cell-dependent antigen responses, switching towards IgG is typically associated with a large decline or disappearance of IgM responses [16]. Different isotypes (IgM, IgG, or IgA) and IgG subclasses (IgG1, IgG2, IgG3, or IgG4) differ in their capacity to recruit immune effector mechanisms. For instance IgM and IgG3 are the most potent complement activators [3]. Likewise, the various IgG subclasses have different affinities to interact with activating or inhibitory Fc receptors [17]. The isotype usage of the ACPA response has been well studied, showing a broad usage of different isotypes by ACPA in RA patients [18–20]. ACPA-IgM and ACPA-IgA are mainly confined to ACPA-IgG-positive patients [18]. Furthermore, an expanded isotype usage of the ACPA response in ACPA-IgG-positive RA is associated with more severe radiographic damage [19]. At present, such information is not available for the anti-CarP antibody response. Various genetic, serologic, and imaging biomarkers have been identified to be associated with radiographic progression in RA [21, 22]. Furthermore, baseline erythrocyte sedimentation rate (ESR) or C-reactive protein (CRP) levels have been reported to associate with radiographic progression; however, these results are not always consistent [22]. However, the presence of more autoantibodies at disease onset associates with increased ESR and CRP levels [23]. For a better understanding of the anti-CarP antibody response, we determined the presence of anti-CarP antibody isotypes and IgG subclasses in baseline serum samples of RA patients with long-term follow-up data in the Leiden Early Arthritis Clinic (EAC) cohort [24]. We show that the anti-CarP antibody response uses a broad spectrum of anti-CarP antibody isotypes and IgG subclasses. Furthermore, the presence of anti-CarP IgG1 associates with more radiographic progression over time and a broader anti-CarP IgG subclass response associates with higher levels of inflammatory markers.

## Methods

### Patients and control sera

The sera analysed were from 373 RA patients (RA1; aged 57.2 ± 15.8 years; 68.1% were female and 58.2% were CCP2-IgG positive; Table 1) and 196 healthy controls (HC; aged 44.1 ± 13.8 years; 50% were female). Anti-CarP antibody IgG data were already available [6] and used in these analyses. Furthermore, as a replication cohort (RA2), 114 RA patients positive for both ACPA and anti-CarP IgG (double-positive) were tested for all ACPA and anti-CarP antibody isotypes and IgG subclasses (aged 54.5 ± 12.01; 63.2% were female and 100% were CCP2-IgG positive; Table 1). The RA patients were participating in the Leiden EAC cohort [24] and were included between 1993 and 2014. Baseline serum samples of these patients were analysed. Healthy control samples were acquired from persons living in the Leiden region as described previously [6]. Most of the RA and HC samples were stored at –20 °C and some at –80 °C; however, previous experiments showed no differences in anti-CarP or ACPA levels and positivity when samples were stored in different ways or after freeze-thawing. Informed consent was obtained from all individuals and all protocols were approved by the ethics committee of the Leiden University Medical Center (LUMC).

**Table 1:** Baseline patient characteristics of the two RA cohorts

	RA1 (*n* = 373)	RA2 (*n* = 114)
Female, *n* (%)	254 (68.1)	72 (63.2)
RF IgM positivity, *n* (%)	225 (60.3)	109 (95.6)
CCP2 IgG positivity, *n* (%)	217 (58.2)	114 (100)
Anti-CarP IgG positivity, *n* (%)	182 (49.2)	114 (100)
“Ever” smokers, *n* (%)	160 (42.9)	75 (65.8)
Age (years), mean (SD)	57.2 (15.8)	54.5 (12.0)

*anti-CarP* anti-carbamylated protein, Ig immunoglobulin, *RA* rheumatoid arthritis, *RA1* discovery cohort, *RA2* replication cohort, *RF* rheumatoid factor, *SD* standard deviation

### Measurement of anti-CarP antibody and ACPA isotypes and IgG subclasses

Anti-CarP antibody isotypes and IgG subclasses were measured by enzyme-linked immunosorbent assay (ELISA) as described previously [6] with some adaptations. Carbamylated fetal calf serum (CaFCS; 10 μg/ml) or non-modified FCS was coated on plates and, after blocking with phosphate-buffered saline (PBS)/1% bovine serum albumin (BSA), serum samples were incubated overnight at 4 °C.

ACPA isotypes and IgG subclasses were measured as described previously [18, 19] with some differences. Briefly, 1 μg/ml CCP2-cittruline or CCP2-arginine was coated on plates and serum samples were incubated for 1 h at 37 °C. The CCP2 peptides were ordered from the peptide facility of the Department of Immunohematology and Blood Transfusion at the LUMC and are provided and produced by Dr. J.W. Drijfhout.

Bound human IgM and IgA was detected using horseradish peroxidase (HRP)-conjugated goat-anti-human (GAH)-IgM (Invitrogen, 627520) or GAH-IgA (Invitrogen, 627420), respectively. For the detection of bound IgG1 and IgG4, HRP-conjugated mouse-anti-human (MAH)-IgG1 (Life Technologies, A10648, Clone HP6069) or MAH-IgG4 (Life Technologies, A10654, Clone HP6025) antibodies were used followed by HRP-conjugated goat-anti-mouse (GAM)-Ig (DAKO, P0447). Bound human IgG2 or IgG3 was detected using MAH-IgG2 (Nordic MUbio, Clone HP6014) or MAH-IgG3 (Nordic MUbio, Clone HP6080) continued with HRP-conjugated GAM-Ig (DAKO, P0447) and rabbit-anti-goat (RAG)-Ig (DAKO, P0449). After the final washings, 2,2′-azino-bis 3-ethylbenzothiazoline-6-sulphonic acid (ABTS) was added to visualize the HRP enzyme activity and the absorbance was measured at 415 nm. To determine the cut-off for a positive response of the anti-CarP antibody isotypes, the mean plus two times the standard deviation was calculated of the specific anti-CarP response in HC [6]. For anti-CarP IgG subclasses the cut-off was set at the 97^th^ percentile of the specific anti-CarP response in HC, as this was equal to what was found using the mean plus two times the standard deviation of total anti-CarP IgG. The upper limits of the anti-CarP antibody ELISAs with the standards used in these experiments are shown in Additional file 1. For ACPA, the cut-off for positivity was defined as the mean plus two times the standard deviation of the measured ACPA response in HC [19].

### Statistical analysis

Statistical analysis was performed using Statistical Package for the Social Sciences (SPSS) version 23 (IBM). In order to determine differences in antibody levels between patients and HC a Mann-Whitney *U* test was carried out. The Pearson chi-squared test was used to determine differences in positivity between RA patients and HC. To investigate whether there are correlations, Spearman rank tests were performed. *P* values below 0.05 were considered statistically significant.

In 373 RA patients the association between anti-CarP antibody isotype or IgG subclass positivity and radiographic progression, as assessed by the Sharp-van der Heijde Score [25], was analysed as described previously [6, 24, 26]. As repeated radiographs were taken at yearly intervals we have used a multivariate normal regression analysis for longitudinal data. Adjustments for treatment strategy, age, sex, baseline ESR, baseline CRP level, and symptom duration have been made. Stratified analysis for ACPA and RF-IgM was performed in 115 RA patients. *P* values below 0.05 were considered statistically significant.

Risk factors for the presence of anti-CarP antibody isotypes and IgG subclasses were investigated by logistic regression analysis. The relation between positivity for anti-CarP antibody isotypes or IgG subclass and CRP levels and ESR were assessed using the Mann-Whitney *U* test. The analysis of the IgG subclasses was performed within the anti-CarP IgG-positive group. Furthermore, the association between the total number of isotypes or IgG subclasses present and risk factors or clinical parameters were investigated by ordinal regression analysis. To correct for multiple testing the Holm-Bonferroni method was applied on independent tests. Since the increasing presence of anti-CCP2 antibody in anti-CarP antibody-positive patients could influence the findings, stratification for anti-CCP2 antibody positivity was performed.

## Results

### The anti-CarP antibody response uses a wide spectrum of isotypes and IgG subclasses

**Fig. 1:**
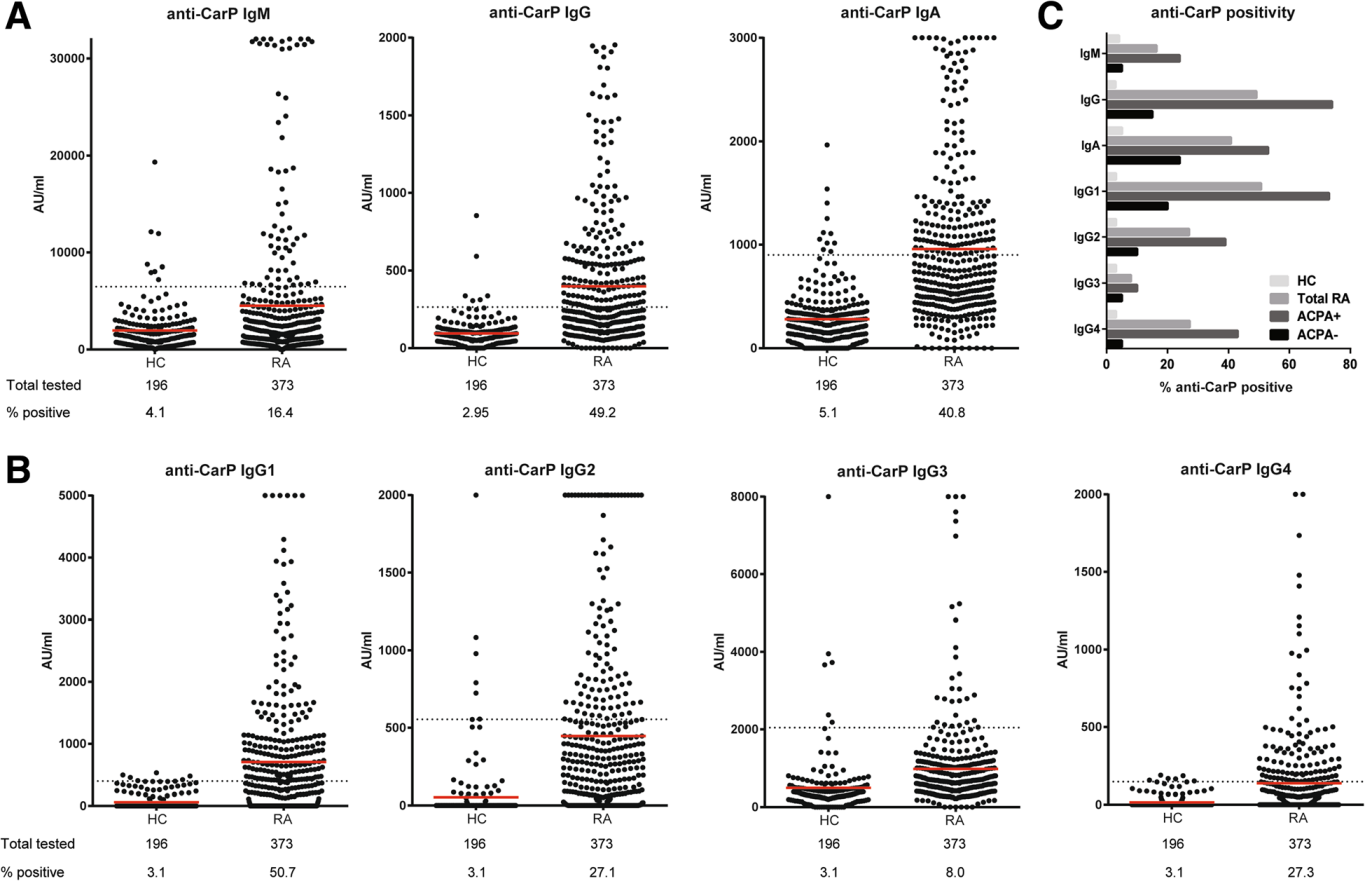
Anti-CarP antibody isotypes and IgG subclasses are present in RA sera. ELISAs were performed to detect anti-carbamylated protein (*anti-CarP*) antibody isotypes (**a**) and immunoglobulin G (*IgG*) subclasses (**b**) in sera of 196 healthy controls (*HC*) and 373 rheumatoid arthritis (*RA*) patients. The mean (*red line*) plus two times the standard deviation in HC was established as the cut-off for the anti-CarP antibody isotypes. The 97^th^ percentile in HC was used as the cut-off for the IgG subclasses. The *dotted line* represents the cut-off. The specific anti-CarP reactivity is depicted in arbitrary units (*AU*) per millilitre. The number of samples tested and the percentage positivity is shown below the graphs. **c** Percentage positivity of anti-CarP antibody isotypes and IgG subclasses in all RA patients (*grey bars*, *n* = 373), anti-citrullinated protein antibody (*ACPA*) IgG-positive RA patients (*dark grey bars*, *n* = 217), ACPA IgG-negative RA patients (*black bars*, *n* = 156), and HC (*light grey bars*, *n* = 196)

Anti-CarP antibody isotypes and IgG subclasses were measured in baseline serum samples of RA patients (*n* = 373) and in serum samples of healthy controls (*n* = 196). In healthy controls, the mean plus two times standard deviation was set as the cut-off, which resulted in positivity for the anti-CarP antibody IgM, IgG, and IgA of 4.1%, 3.0% (results from Shi et al. [6]), and 5.1%, respectively, in the control population. At this cut-off we observed that 16.4% of the RA patients were positive for IgM anti-CarP antibodies, while 49.2% and 40.8% were positive for IgG (part of the results from Shi et al. [6]) and IgA (Fig. 1a). Furthermore, reactivity for all anti-CarP IgG subclasses was analysed in healthy controls and RA patients. The results of these analyses show that 50.7% of the RA patients are positive for anti-CarP IgG1, 27.1% for IgG2, 8.0% for IgG3, and 27.3% for IgG4 (Fig. 1b). When comparing levels or percent-positivity between healthy controls and RA patients, significant differences (*p* < 0.001 for all analyses) were observed for all anti-CarP antibody isotypes and IgG subclasses analysed. Overall, these data indicate that the anti-CarP antibody response is characterized by wide usage of anti-CarP antibody isotypes and IgG subclasses in baseline serum samples of RA patients.

### Presence of anti-CarP antibody isotypes and IgG subclasses in ACPA-positive and ACPA-negative disease

The anti-CarP antibodies of the IgG isotype are found in approximately 15% of ACPA-negative RA patients but are mainly detected in ACPA-positive RA patients [6]. We therefore investigated the anti-CarP antibody isotype and IgG subclass distribution in ACPA-positive and -negative RA. Interestingly, all anti-CarP antibody isotypes and IgG subclasses could be detected in ACPA-negative disease (5, 15, and 24% positive for the anti-CarP antibody IgM, IgG, and IgA, and 20, 10, 5, and 5% positive for anti-CarP antibody IgG1, IgG2, IgG3, and IgG4, respectively; Fig. 1c). However, no significant differences were found for anti-CarP antibody IgM, IgG3, and IgG4 between RA patients and HC at this group size. For ACPA-positive RA, a higher proportion of patients were positive for anti-CarP antibodies with 24, 74, and 53% positive for the IgM, IgG, and IgA, and 73, 39, 10, and 43% positive for IgG1, IgG2, IgG3, and IgG4, respectively (Fig. 1c). Significant differences were found for all anti-CarP antibody isotypes and IgG subclasses between RA patients and HC.

Thus, together, all anti-CarP antibody isotypes and IgG subclasses analysed can be detected in sera of RA patients with the highest number of patients positive for anti-CarP antibodies in ACPA-positive disease.

### The numbers of anti-CarP antibody isotypes and IgG subclasses are related to anti-CarP levels

**Fig. 2:**
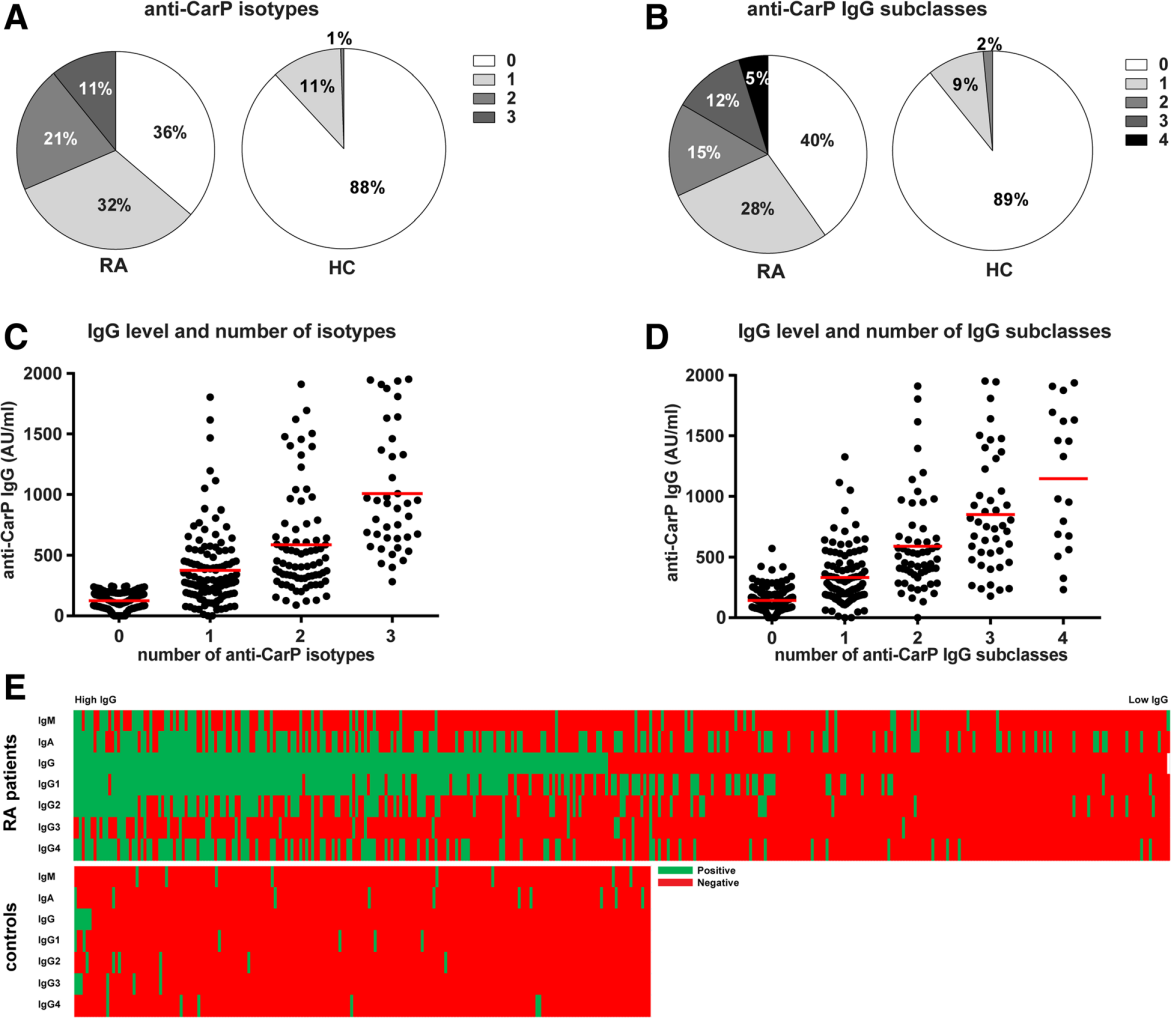
The number of anti-CarP antibody isotypes and IgG subclasses differs between RA patients and is level dependent. Anti-carbamylated protein (*anti-CarP*) antibody isotypes and immunoglobulin G (*IgG*) subclasses were measured by ELISA in 373 rheumatoid arthritis (*RA*) patients and 196 healthy controls (*HC*). Pie charts show the percentage of RA patients and HC negative or positive for one, two, or three anti-CarP antibody isotypes (**a**) and negative or positive for one, two, three, or four anti-CarP IgG subclasses (**b**). An increase in level of anti-CarP antibody IgG associates with an increase in the number of anti-CarP antibody isotypes (**c**) and IgG subclasses (**d**) in RA patients. *Red lines* depict means (**e**). Heat maps show the presence of anti-CarP antibody isotypes and IgG subclasses in RA and HC, ranked according to anti-CarP antibody IgG levels. *Green* and *red* mark positive and negative sera, respectively. *AU* arbitrary units

Because a wide repertoire of anti-CarP antibody subtypes was identified, we next determined how many different anti-CarP antibody isotypes or IgG subclasses are present in sera of RA patients. Of all tested RA patients, 36% were negative for all anti-CarP antibody isotypes (Fig. 2a). Of the anti-CarP antibody-positive patients, most expressed one isotype (32%), whereas 21% and 11% expressed two or three isotypes, respectively (Fig. 2a). Furthermore, anti-CarP antibody IgG subclasses were detected in 60% of the RA patients; 28% expressed one IgG subclass, 15% two subclasses, 12% three subclasses, and 5% expressed all four subclasses (Fig. 2b). For healthy controls, 88% tested negative for all isotypes analysed, 11% was positive for one isotype, 1% for two isotypes, and none of the healthy controls were positive for all three isotypes (Fig. 2a). Similar percentages of positivity were found for the number of IgG subclasses in healthy controls (Fig. 2b). These data indicate that the anti-CarP antibody response is not uniform and that considerable variation can exist between anti-CarP antibody-positive patients with respect to the depth of the anti-CarP antibody response.

In addition, we investigated whether a correlation could be observed between the anti-CarP antibody IgG level and the number of anti-CarP antibody isotypes or IgG subclasses present in RA patients. As depicted in Fig. 2c, the number of anti-CarP antibody isotypes in RA patients was associated with anti-CarP IgG level (correlation coefficient is 0.748, *p* < 0.001), as was the number of anti-CarP IgG subclasses (correlation coefficient 0.75, *p* < 0.001) (Fig. 2d).

An overview of anti-CarP antibody isotype and IgG subclass expression by RA patients is depicted in a heatmap, ranked according to anti-CarP IgG antibody level (Fig. 2e). Interestingly, some of the RA patients tested positive for anti-CarP IgM and/or IgA, although they were negative for anti-CarP antibody IgG.

Altogether, these data indicate that the anti-CarP antibody response differs between individual RA patients and that higher anti-CarP antibody levels are associated with the presence of more anti-CarP antibody isotypes and IgG subclasses.

### Distribution of anti-CarP antibody and ACPA isotypes and IgG subclasses

**Fig. 3:**
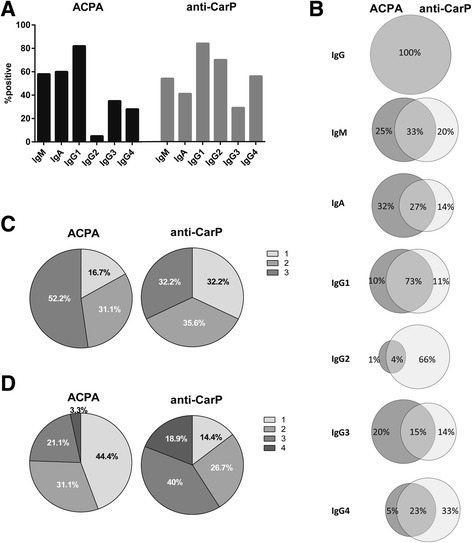
Distribution of anti-CarP and ACPA isotypes and IgG subclasses. **a** Percentage positivity of anti-citrullinated protein antibody (*ACPA*; *black bars*) or anti-carbamylated protein (*anti-CarP*) antibody (*grey bars*) immunoglobulin (*Ig*)M, IgA, and IgG subclasses in ACPA and anti-CarP antibody IgG double-positive RA patients (*n* = 114). **b** Percentage single- or double-positive for ACPA (*grey*) and anti-CarP antibody (*light grey*) isotypes and IgG subclasses in IgG double-positive RA patients. Circles are not to scale. Number of anti-CarP antibody and ACPA isotypes (**c**) and IgG subclasses (**d**) in anti-CarP antibody and ACPA IgG double-positive RA patients and at least positive for one IgG subclass (*n* = 90)

To investigate whether the anti-CarP antibody and ACPA response displays a similar usage of isotypes and IgG subclasses, the presence of anti-CarP isotypes and IgG subclasses was studied within anti-CarP antibody and ACPA IgG double-positive RA patients. We observed a similar distribution of several isotypes and subclasses, although for others a different distribution was noted for both autoantibody responses at the population level (Fig. 3a and b and Additional file 2). Approximately half of the patients tested positive for ACPA IgM or IgA. Similar results were observed for the anti-CarP IgM and IgA antibody. When investigating IgG subclasses, almost 80% of the patients were positive for IgG1 ACPA or IgG1 anti-CarP antibody and around 40% of the patients showed positivity for ACPA or anti-CarP antibody IgG3. Interestingly, a considerable difference between both autoantibody reactivities was seen for IgG2, as 5% of the patients tested positive for ACPA IgG2, whereas 70% of the patients were positive for anti-CarP antibody IgG2. For IgG4, 28% of the patients were positive for IgG4 ACPA and 56% of the patients for IgG4 anti-CarP antibody. As depicted in Fig. 3b, patients positive for a particular isotype or IgG subclass group could test positive for both autoantibodies in the same isotype/IgG subclass, but discordance is also observed frequently. Furthermore, a weak correlation was observed between the anti-CarP antibody and ACPA response for each isotype or IgG subclass. In the same sample set we observed a more pronounced correlation between anti-CarP IgM and RF-IgM as well as ACPA IgM and RF-IgM (Additional file 3). We next investigated the correlation between anti-CarP and ACPA in an unselected group of RA patients (also including ACPA/anti-CarP-negative patients) to further confirm these findings. In agreement with the data presented above, we again observed weak correlations for anti-CarP and ACPA (Additional file 4). When we examined the number of ACPA and anti-CarP antibody isotypes or IgG subclasses present in the patient population, similar results were observed for both antibody responses. However, around half of the ACPA IgG-positive patients were positive for three isotypes, whereas this was the case in only 32.2% of anti-CarP antibody-positive patients (Fig. 3c). Furthermore, approximately half of the ACPA IgG-positive patients tested positive for only one ACPA IgG subclass, whereas this was the case in only 14% of anti-CarP IgG-positive patients (Fig. 3d). No association was found between the number of ACPA isotypes/IgG subclasses and anti-CarP antibody isotypes/IgG subclasses used by the respective autoantibody responses (data not shown).

Although at the population level the usage of most isotypes/IgG subclasses was similar for both autoantibody reactivities it was different for other isotypes/IgG subclasses. The latter was most prominent within individual patients as they could be single- or double-positive for the anti-CarP and/or ACPA isotype/IgG subclass. Together, these data indicate that the expression of these two autoantibody responses is differentially regulated.

### Anti-CarP IgG1 antibodies are associated with more severe radiological damage, also in ACPA- and RF-negative RA

**Fig. 4:**
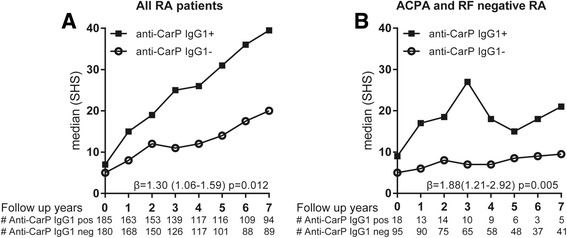
Anti-CarP IgG1 is associated with more severe radiological progression. The extent and rate of joint destruction was analysed in all rheumatoid arthritis (*RA*) patients or separately for anti-citrullinated protein antibody (*ACPA*)-negative and ACPA-positive RA and within the ACPA-negative RA patients also separately for rheumatoid factor (RF) negative and positive. The severity of joint damage is depicted as median Sharp-van der Heijde score (*SHS*) on the *y* axis and the follow-up years on the *x* axis for anti-carbamylated protein (*anti-CarP*) antibody immunoglobulin (*Ig*)G1-positive and -negative patients in all RA patients analysed (**a**) and for ACPA- and RF-negative RA patients (**b**). β and *p* values are derived from the analysis model as described in the Methods and Results sections

Previous data have shown that the presence of anti-CarP IgG antibodies are associated with more severe radiological progression, and also after correction for ACPA and RF stratification in ACPA-positive and ACPA-negative RA [6, 9, 10, 12]. Likewise, a similar trend has been observed for ACPA-negative patients positive for anti-CarP IgA [6]. To analyse whether the presence of anti-CarP IgM, IgA, and anti-CarP IgG subclasses is also predictive for a more severe disease course, we compared the extent of joint damage over time, measured with the Sharp-van der Heijde method, between anti-CarP antibody-positive and -negative patients (Fig. 4). The calculated β indicates the rate of joint destruction after 7 years compared to the reference group after adjustments for treatment strategy, age, sex, baseline ESR, baseline CRP, and symptom duration [24, 26]. Anti-CarP IgG1-positive patients displayed more joint destruction over 7 years than anti-CarP IgG1-negative patients without correction for ACPA and RF (β = 1.30, 95% confidence interval (CI) 1.06–1.59, *p* = 0.012; Fig. 4a). Stratified analyses were performed for ACPA alone (corrected for RF, data not shown) and ACPA and RF together. Importantly, this analysis revealed that the presence of anti-CarP IgG1 in ACPA- and RF-negative RA associates with more severe joint destruction over 7 years (β = 1.88, 95% CI 1.21–2.92, *p* = 0.005) (Fig. 4b).

Next to anti-CarP IgG1, anti-CarP IgG4 antibody positivity was found to be associated with more joint damage over 7 years (β = 1.24, 95% CI 1.00–1.53, *p* = 0.045); however, this correlation was lost after correction for ACPA (*p* = 0.39) or after stratification for ACPA status (*p* = 0.28). Furthermore, no significant associations with joint damage were found for anti-CarP IgG2, IgG3, IgM, and IgA antibodies. Moreover, no associations with severity were found with a broader anti-CarP antibody isotype or IgG subclass usage.

Taken together, these data indicate that the detection of anti-CarP IgG1 at baseline is predictive for a more severe disease course in ACPA-negative (data not shown) and ACPA- and RF-negative RA (Fig. 4b).

### Broader IgG subclass usage associates with higher erythrocyte sedimentation rate and higher C-reactive protein levels

To study the relationship between anti-CarP antibody isotypes or IgG subclasses and baseline characteristics, we next analysed the association between the number of anti-CarP isotypes or IgG subclasses and ESR or CRP level at onset using ordinal regression analyses. This showed an association between the number of anti-CarP IgG subclasses and increased ESR and CRP level (Table 2), especially in ACPA-positive disease (*p* = 0.001 for both which remained significant after Holm-Bonferroni correction, data not shown). When analysing the anti-CarP IgG subclasses separately in the anti-CarP total IgG-positive group, the association with increased ESR and CRP is mostly manifested between the anti-CarP antibody IgG4-positive and -negative patients (*p* < 0.001 for ESR and *p* = 0.003 for CRP), also after Holm-Bonferroni correction (Table 3 and Additional file 5) and after stratification in the ACPA-positive group (data not shown).

**Table 2:** Associations for the number of anti-carbamylated protein antibody isotypes or IgG subclasses with risk factors and baseline characteristics

Number of isotypes	0 (*n* = 135)	1 (*n* = 120)	2 (*n* = 77)	3 (*n* = 40)		Ordinal OR (95% CI)	*p*
Smoking (ever), *n* (%)	54 (42.9)	48 (43.6)	35 (48.6)	23 (60.5)		1.38 (0.94-2.03)	0.099
SE positivity, *n* (%)	79 (60.3)	74 (63.2)	57 (75.0)	33 (86.8)		**1.91 (1.27-2.87)**	0.002*
ESR (mm/h), mean ± SD	41.0 ± 25.6	40.2 ± 25.1	43.5 ± 27.7	47.8 ± 26.8		1.01 (1.00-1.01)	0.206
CRP (mg/L), mean ± SD	29.0 ± 32.2	32.3 ± 36.4	29.9 ± 30.3	50.8 ± 47.0		**1.01 (1.00-1.01)**	**0.017** ^**‡**^
Number of IgG subclasses	0 (*n* = 150)	1 (*n* = 104)	2 (*n* = 57)	3 (*n* = 44)	4 (*n* = 18)	Ordinal OR (95% CI)	*p*
Smoking (ever), *n* (%)	58 (41.1)	42 (42.4)	30 (58.8)	27 (65.9)	3 (20.0)	1.47 (1.00-2.16)	0.051^‡^
SE positivity, *n* (%)	89 (60.5)	65 (64.4)	46 (80.7)	31 (75.6)	13 (76.5)	**1.79 (1.19-2.69)**	0.005*
ESR (mm/h), mean ± SD	37.9 ± 23.2	37.7 ± 24.3	50.4 ± 31.1	52.3 ± 28.5	48.8 ± 21.6	**1.01 (1.01-1.02)**	<0.001*
CRP (mg/L), mean ± SD	30.5 ± 32.9	28.1 ± 33.9	30.5 ± 34.8	45.4 ± 41.5	50.2 ± 42.9	**1.01 (1.00-1.01)**	**0.020**

Ordinal regression analysis for the number of isotypes present and risk factors (smoking, SE positivity) or baseline parameters (ESR, CRP level)

Bold text indicates a significant difference

* Remained significant after Holm-Bonferroni correction

^‡^ Test of parallel lines significant

Holm-Bonferroni correction was performed for independent analyses in the whole cohort

*CI* confidence interval, *CRP* C-reactive protein, *ESR* erythrocyte sedimentation rate, *IgG* immunoglobulin G, *OR* odds ratio, *SD* standard deviation, *SE* shared epitope

**Table 3:** Associations of anti-carbamylated protein antibody isotypes and IgG subclasses with baseline characteristics

Isotypes	IgG– (*n* = 190)	IgG + (*n* = 182)	*p*	IgA– (*n* = 221)	IgA + (*n* = 152)	*p*	IgM– (*n* = 312)	IgM + (*n* = 61)	*p*			
ESR, mm/h			0.676			0.157			0.216			
Mean ± SD Median (IQR)	41.2 ± 25.3 37.0 (33.3)	42.9 ± 26.8 37.0 (44.5)		40.4 ± 25.7 34.5 (33.0)	44.2 ± 26.4 39.0 (43.0)		41.2 ± 25.8 37.0 (35.0)	45.7 ± 26.8 44.0 (45.0)				
CRP, mg/L			0.694			**0.039**			0.092			
Mean ± SD Median (IQR)	32.0 ± 35.7 19.0 (34.0)	33.3 ± 35.4 19.5 (36.3)		28.9 ± 33.3 17.0 (28.8)	38.0 ± 38.0 23.0 (48.0)		29.9 ± 31.8 18.0 (31.0)	46.0 ± 48.3 23.5 (59.8)				
IgG subclasses	IgG1– (*n* = 33)	IgG1 + (*n* = 149)	*p*	IgG2– (*n* = 100)	IgG2 + (*n* = 82)	*p*	IgG3– (*n* = 157)	IgG3 + (*n* = 25)	*p*	IgG4– (*n* = 98)	IgG4 + (*n* = 84)	*p*
ESR, mm/h			0.176			0.156			0.323			<0.001*
Mean ± SD Median (IQR)	36.8 ± 24.2 32.0 (26.0)	44.1 ± 27.3 37.0 (46.0)		40.4 ± 26.3 34.0 (39.0)	45.7 ± 27.4 44.5 (46.3)		42.1 ± 26.9 34.5 (45.0)	47.0 ± 26.6 51.0 (45.0)		35.3 ± 23.2 30.0 (29.3)	51.4 ± 28.3 49.0 (45.0)	
CRP, mg/L			0.637			**0.023**			0.107			0.003*
Mean ± SD Median (IQR)	27.5 ± 26.1 20.0 (30.0)	34.7 ± 37.2 19.5 (39.3)		29.0 ± 33.1 17.5 (31.8)	38.7 ± 37.7 26.0 (41.0)		31.1 ± 33.4 18.0 (33.5)	47.5 ± 44.8 32.0 (58.5)		25.3 ± 27.2 16.0 (28.0)	42.1 ± 41.1 28.0 (52.0)	

Mann-Whitney *U* test to calculate *P* values for baseline parameters (ESR and CRP level)

IgG subclass analysis was performed in anti-carbamylated protein IgG-positive patients

Bold indicates a significant difference

* Remained significant after Holm-Bonferroni correction

Holm-Bonferroni correction was performed for independent analyses in whole cohort

*CRP* C-reactive protein, *ESR* erythrocyte sedimentation rate, *Ig* immunoglobulin, *IQR* interquartile range, *SD* standard deviation

To summarize, these data indicate that a broader anti-CarP IgG subclass response associates with increased ESR and CRP level in ACPA-positive disease.

## Discussion

In this study, we analysed the presence and levels of anti-CarP antibody isotypes and IgG subclasses in patients with RA. The results indicate that the anti-CarP antibody response uses a broad spectrum of isotypes and IgG subclasses in RA patients. The presence of anti-CarP antibody IgM together with other isotypes might indicate that there is an ongoing immune response which is continuously reactivated by IgM producing B cells. This is because IgM antibodies have a short half-life of a few days [27] and, in the presence of T cell help, switching towards IgG is typically associated with the disappearance of the IgM responses [16].

In the overall RA group (*n* = 373), as well as in the ACPA-negative subgroup, a higher percentage of RA patients tested positive for anti-CarP IgG1 than for total anti-CarP IgG. This apparent discrepancy can likely be explained best by the sensitivities of these different assays in combination with the cut-offs used to define a positive response. For example, the total IgG anti-CarP ELISA measures the presence of all four IgG subclasses, whereas the IgG subclass ELISA detects only one of them. The cut-offs for each of these assays were calculated separately, based on the measurement in healthy controls. Therefore, patients could be tested positive when measuring only IgG1 and not when analysed for total anti-CarP IgG (the “sum” of IgG1, IgG2, IgG3, and IgG4).

A limitation of the study is that the HC and RA patients were not completely age and sex matched. The HC group was used to set the cut-off for anti-CarP and anti-CCP positivity and it was considered more important to use a control group that is reflecting the overall population of the area in which the disease population is living. Importantly, we do not have indications that the presence of anti-CarP antibody and ACPA in HC is associated with gender, age, or smoking.

To study whether there was a similar distribution of ACPA and anti-CarP antibody isotype usage in IgG double-positive RA patients, 114 double-positive RA patients were also tested. We observed that the distribution of isotype and IgG subclass usage and the number of isotypes and IgG subclasses expressed is similar for both autoantibodies at the population level. However, within individual patients this pattern could be different for anti-CarP and ACPA. This is of interest as it indicates that it is not merely a reflection of a cross-reactive antibody. These observations are further strengthened by the absence of a clear correlation between the anti-CarP and ACPA isotypes and IgG subclasses, a correlation that was found for the presence of anti-CarP IgM or ACPA IgM, respectively, and RF-IgM. Previously we analysed cross-reactivity between ACPA and anti-CarP antibodies in a dedicated set-up and observed both cross-reactive and non-cross-reactive antibody recognition profiles [28].

In previous studies, it has been shown that RA patients who are positive for anti-CarP antibody IgG display more radiological damage over time compared to anti-CarP antibody IgG-negative patients, also when corrected for ACPA and RF [6, 12]. This study emphasizes this notion since the presence of anti-CarP IgG1 antibodies is associated with more joint damage, even after correction and stratification for ACPA and RF. However, measuring one of the IgG subclasses did not out-perform the measurement of total IgG as a predictor for joint damage. Furthermore, no added value was observed for measuring anti-CarP antibody IgG1 instead of total IgG. The observation that none of the other IgG subclasses or isotypes provided significant associations with severity could be because the number of individuals positive for the other IgG subclasses and isotypes is lower which results in a decreased statistical power to detect an association. Although we do find an association between anti-CarP IgG1 positivity and severity using correction for possible confounders (age, sex, treatment, baseline ESR, baseline CRP, and symptom duration) and after stratification for ACPA and RF, it is difficult to separate out all possible confounders. Likewise, subdividing the group into all possible subgroups will certainly decrease the sample size, leading to a further loss in power.

When investigating the number of anti-CarP antibody isotypes or IgG subclasses used, no clear associations were found with joint damage. However, a broader anti-CarP IgG subclass usage was associated with an increase in inflammatory markers (higher ESR and CRP level) at baseline which was mostly manifested in anti-CarP IgG4. The percentage of ACPA-positive patients was similar in the groups that expressed more than one anti-CarP IgG subclass. However, the ACPA levels are higher in patients with more anti-CarP antibody IgG subclasses. This could be explained by the fact that the number of anti-CarP antibody IgG subclasses associates with higher anti-CarP antibody levels and higher anti-CarP antibody levels are associated with higher ACPA and RF levels, and vice versa.

In the current study, we have analysed the presence of anti-CarP antibody isotypes and IgG subclasses in patients diagnosed with early RA. It would be interestingly to study the pre-RA phase as well to see how the distribution of anti-CarP antibody isotypes and IgG subclasses develops over time.

## Conclusion

In conclusion, the anti-CarP antibody response uses a broad spectrum of isotypes and IgG subclasses, indicating an ongoing immune response and ample possibilities to employ several effector mechanisms. Furthermore, the anti-CarP and ACPA autoantibody responses seem to be differentially regulated.

## Additional files

Additional file 1: Upper limit of the anti-CarP antibody ELISAs. (PDF 243 kb)

Additional file 2: Presence of anti-CarP antibody and ACPA isotypes and IgG subclasses in IgG double-positive patients. ELISAs were performed to detect anti-CarP antibody and ACPA isotypes and IgG subclasses in sera of 80 HC and 114 RA patients. The mean plus two times the standard deviation in HC was established as the cut-off for the anti-CarP antibody isotypes and for the ACPA isotypes and IgG subclasses. The 97^th^ percentile in HC was used as cut-off for the anti-CarP antibody IgG subclasses. Dotted line represents cut-off. The specific anti-CarP antibody reactivity, FCS reactivity subtracted from the CaFCS reactivity, is depicted in AU/ml (A). For ACPA, the reactivity for CCP2-cittruline is depicted in AU/ml (B) and the % positivity was corrected for reactivity against CCP2-arginine. The amount of samples tested and the percentage positivity is shown below the graphs. HC; healthy controls, RA; rheumatoid arthritis, ACPA; anti-citrullinated protein antibodies, anti-CarP antibody ; anti-carbamylated protein antibody, FCS; fetal calf serum, CaFCS; carbamylated fetal calf serum, AU/ml; arbitrary units per millilitre. (TIF 25721 kb)

Additional file 3: Correlation of anti-CarP antibody and ACPA IgM, IgA, and IgG subclasses and RF-IgM with anti-CarP antibody and ACPA IgM in IgG double-positive RA. ELISAs were performed to detect anti-CarP antibody and ACPA isotypes and IgG subclasses in sera of 114 RA patients. Levels of anti-CarP antibodies and ACPAs were plotted against each other, each IgG subclass and isotype separately (A–F). As internal control anti-CarP IgM and ACPA IgM were plotted against RF-IgM (G, H). Spearman Rank tests were performed to investigate correlations. HC; healthy controls, RA; rheumatoid arthritis, ACPA; anti-citrullinated protein antibodies, anti-CarP antibody; anti-carbamylated protein antibody, RF; rheumatoid factor, AU/ml; arbitrary units per millilitre. (TIF 42723 kb)

Additional file 4: Presence and correlation of anti-CarP antibody and ACPA IgM, IgG, IgA, and IgG subclasses and correlations of RF-IgM with anti-CarP antibody and ACPA IgM in an unselected group of RA patients. ELISAs were performed to detect anti-CarP antibody and ACPA isotypes and IgG subclasses in sera of 149 unselected RA patients. ACPA and anti-CarP antibody isotype and IgG subclass positivity, percentage and numbers (A). Levels of anti-CarP antibodies and ACPAs were plotted against each other, each isotype and IgG subclass separately (B–H). As internal control anti-CarP IgM and ACPA IgM were plotted against RF-IgM (I, J). Spearman Rank tests were performed to investigate correlations. HC; healthy controls, RA; rheumatoid arthritis, ACPA; anti-citrullinated protein antibodies, anti-CarP antibody; anti-carbamylated protein antibody, RF; rheumatoid factor, AU/ml; arbitrary units per millilitre. (TIF 55961 kb)

Additional file 5: Associations of anti-CarP antibody isotypes and IgG subclasses with risk factors. Logistic regression univariate for risk factor analysis (smoking and SE positivity) IgG subclass analysis in anti-CarP IgG positive patients. Bold indicates a significant difference. * Remained significant after Holm-Bonferroni correction. Holm-Bonferroni correction separately for independent analyses in whole cohort. SE, shared epitope; OR, odds ratio; CI, confidence interval. (PDF 91 kb)

## Abbreviations

ABTS: 2,2′-Azino-bis 3-ethylbenzothiazoline-6-sulphonic acid
ACPA: Anti-citrullinated protein antibody
anti-CarP: Anti-carbamylated protein
CaFCS: Carbamylated fetal calf serum
CI: Confidence interval
CRP: C-reactive protein
EAC: Early Arthritis Clinic
ELISA: Enzyme-linked immunosorbent assay
ESR: Erythrocyte sedimentation rate
FCS: Fetal calf serum
GAH: Goat-anti-human
GAM: Goat-anti-mouse
HC: Healthy controls
HRP: Horseradish peroxidase
Ig: Immunoglobulin
MAH: Mouse-anti-human
RA: Rheumatoid arthritis
RF: Rheumatoid factor
SF: Synovial fluid
SPSS: Statistical Package for the Social Sciences
