# Supplementary material for: The isotype and IgG subclass distribution of anti-carbamylated protein antibodies in rheumatoid arthritis patients
Source: Arthritis Res Ther. 2017 Aug 15;19:190. doi: 10.1186/s13075-017-1392-z (PMC5558706; doi:10.1186/s13075-017-1392-z)
Supplement: Supplementary file 1 — Upper limit of the anti-CarP antibody ELISAs. (PDF 243 kb) [file 13075_2017_1392_MOESM1_ESM.pdf]

Upper limit of the anti-CarP antibody ELISAs

Anti-CarP Isotype/ IgG-subclass	Upper limit (AU/ml)
IgM	32,000
IgG	2,000
IgA	3,160
IgG1	10,200
IgG2	2,000
IgG3	8,000
IgG4	2,000
