# Supplementary figures and images for: The isotype and IgG subclass distribution of anti-carbamylated protein antibodies in rheumatoid arthritis patients
Source: Arthritis Res Ther. 2017 Aug 15;19:190. doi: 10.1186/s13075-017-1392-z (PMC5558706; doi:10.1186/s13075-017-1392-z)

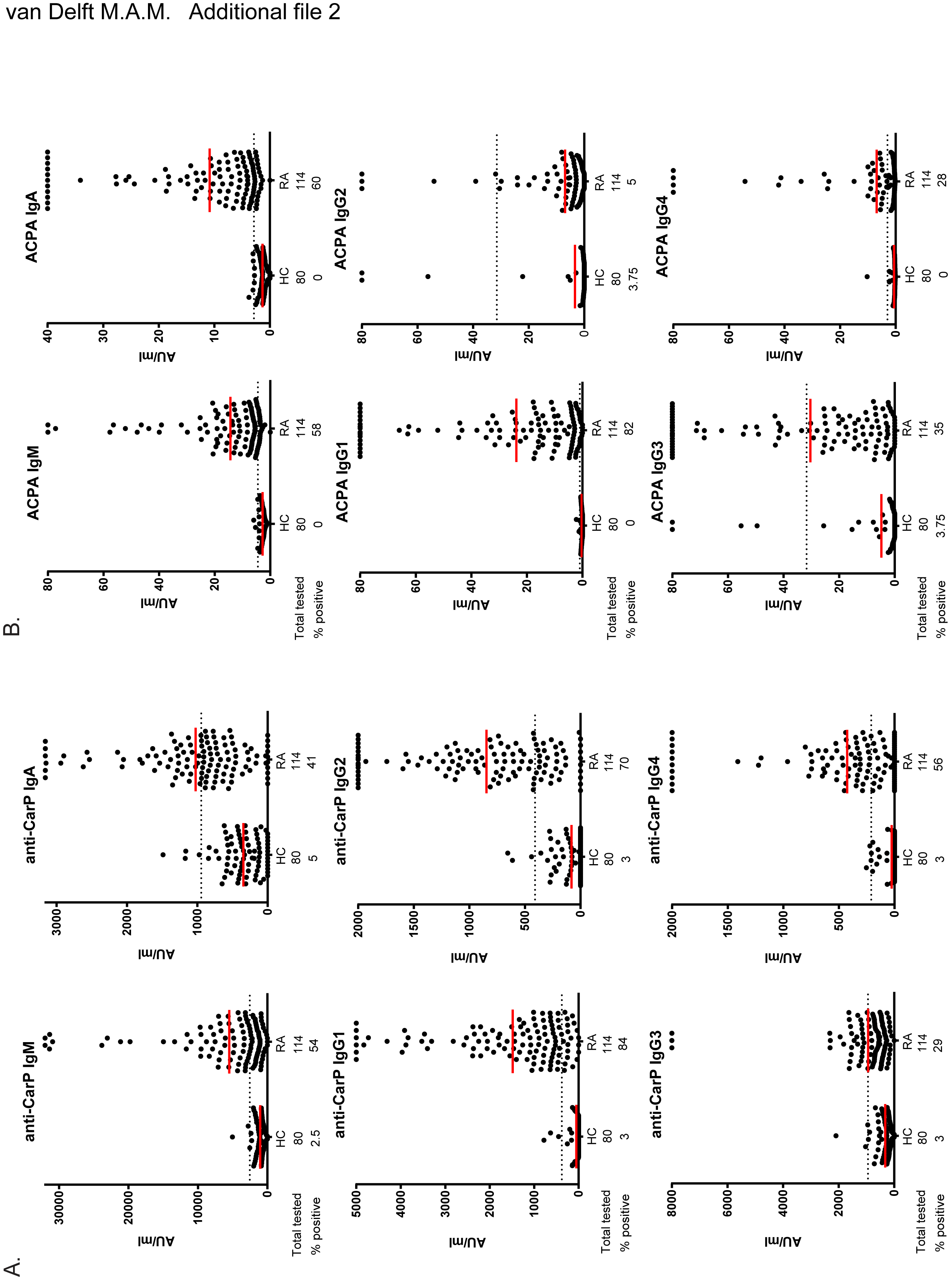

Supplement: Supplementary file 2 — Presence of anti-CarP antibody and ACPA isotypes and IgG subclasses in IgG double-positive patients. ELISAs were performed to detect anti-CarP antibody and ACPA isotypes and IgG subclasses in sera of 80 HC and 114 RA patients. The mean plus two times the standard deviation in HC was established as the cut-off for the anti-CarP antibody isotypes and for the ACPA isotypes and IgG subclasses. The 97th percentile in HC was used as cut-off for the anti-CarP antibody IgG subclasses. Dotted line represents cut-off. The specific anti-CarP antibody reactivity, FCS reactivity subtracted from the CaFCS reactivity, is depicted in AU/ml (A). For ACPA, the reactivity for CCP2-cittruline is depicted in AU/ml (B) and the % positivity was corrected for reactivity against CCP2-arginine. The amount of samples tested and the percentage positivity is shown below the graphs. HC; healthy controls, RA; rheumatoid arthritis, ACPA; anti-citrullinated protein antibodies, anti-CarP antibody ; anti-carbamylated protein antibody, FCS; fetal calf serum, CaFCS; carbamylated fetal calf serum, AU/ml; arbitrary units per millilitre. (TIF 25721 kb) [file 13075_2017_1392_MOESM2_ESM.tif]

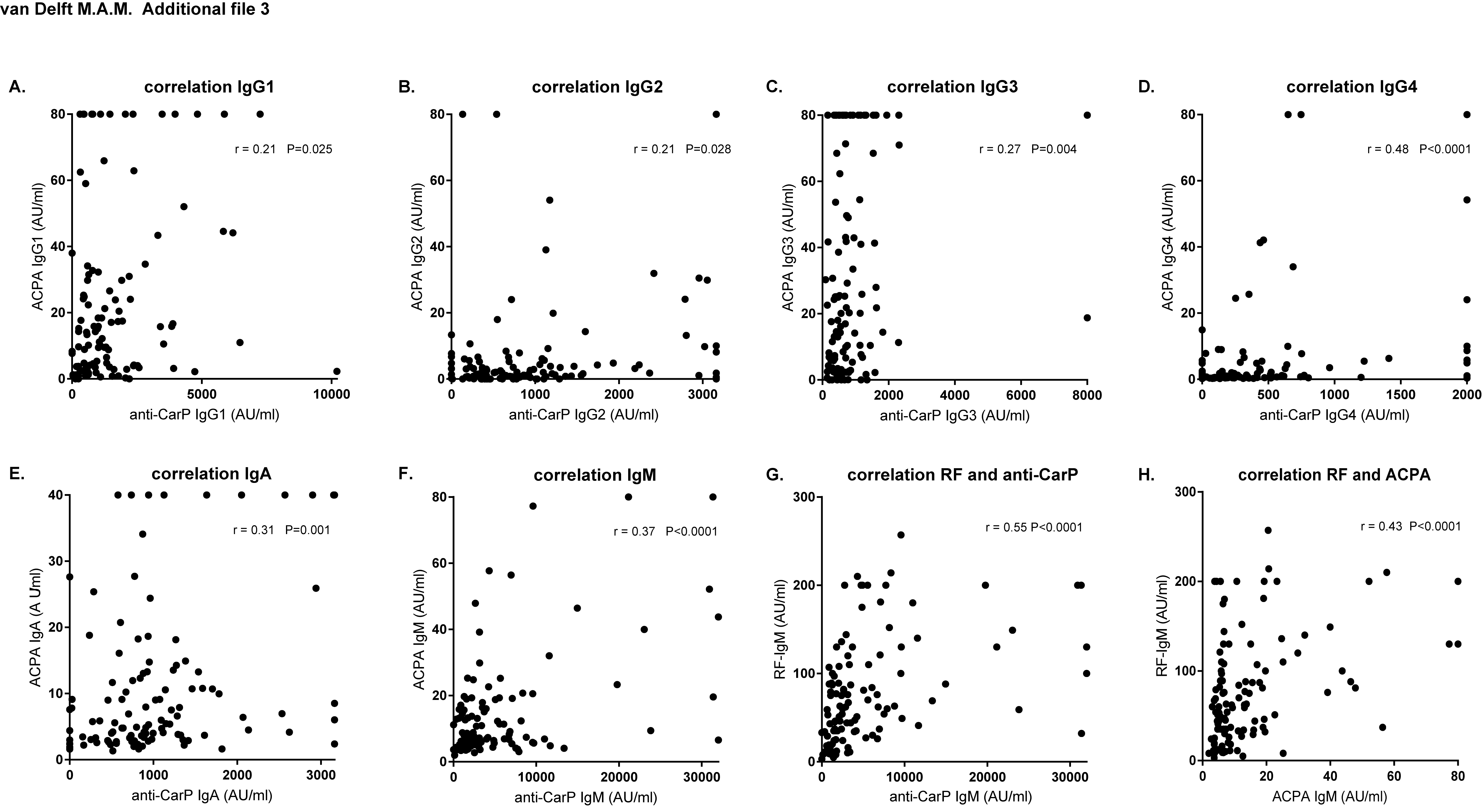

Supplement: Supplementary file 3 — Correlation of anti-CarP antibody and ACPA IgM, IgA, and IgG subclasses and RF-IgM with anti-CarP antibody and ACPA IgM in IgG double-positive RA. ELISAs were performed to detect anti-CarP antibody and ACPA isotypes and IgG subclasses in sera of 114 RA patients. Levels of anti-CarP antibodies and ACPAs were plotted against each other, each IgG subclass and isotype separately (A–F). As internal control anti-CarP IgM and ACPA IgM were plotted against RF-IgM (G, H). Spearman Rank tests were performed to investigate correlations. HC; healthy controls, RA; rheumatoid arthritis, ACPA; anti-citrullinated protein antibodies, anti-CarP antibody; anti-carbamylated protein antibody, RF; rheumatoid factor, AU/ml; arbitrary units per millilitre. (TIF 42723 kb) [file 13075_2017_1392_MOESM3_ESM.tif]

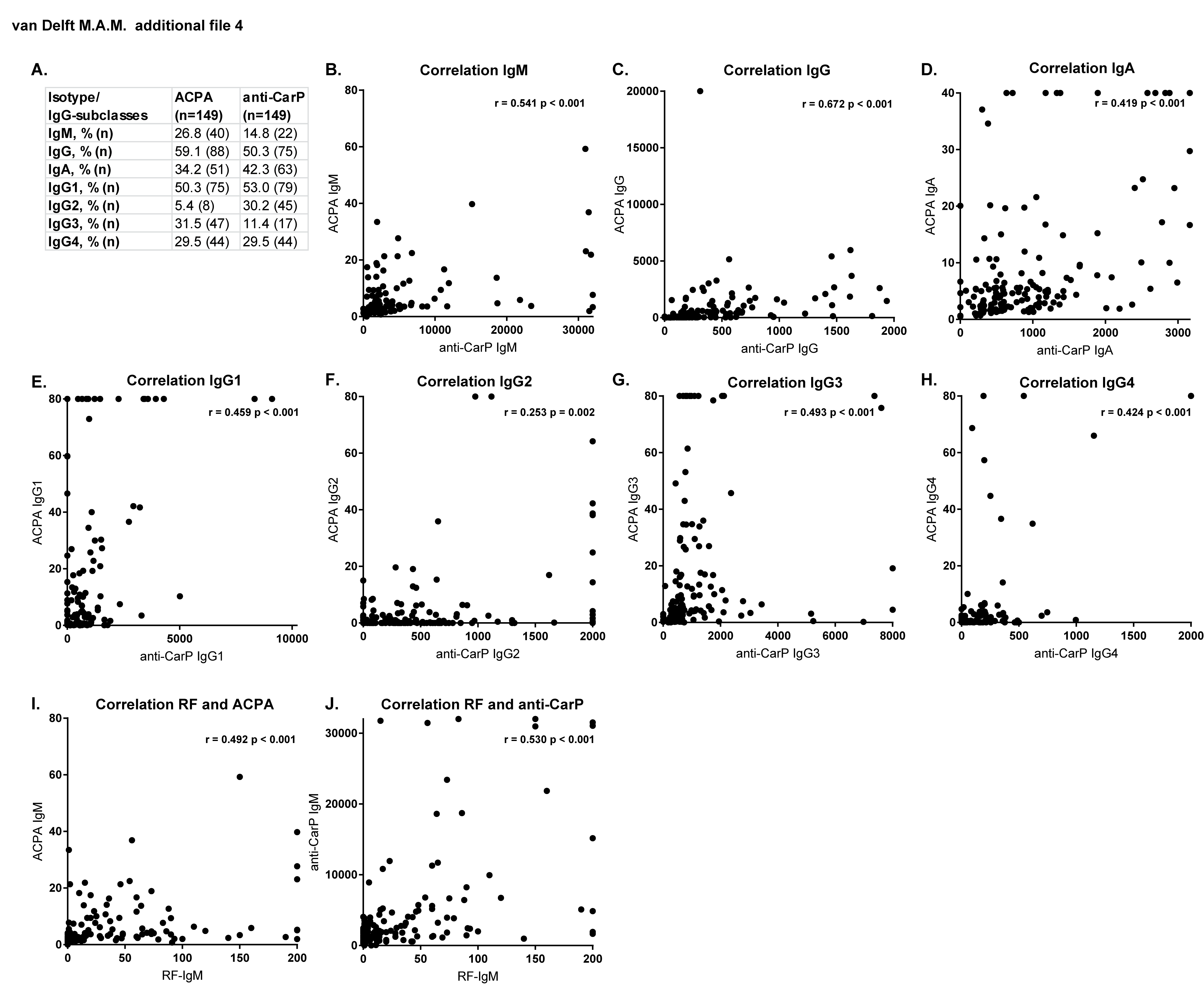

Supplement: Supplementary file 4 — Presence and correlation of anti-CarP antibody and ACPA IgM, IgG, IgA, and IgG subclasses and correlations of RF-IgM with anti-CarP antibody and ACPA IgM in an unselected group of RA patients. ELISAs were performed to detect anti-CarP antibody and ACPA isotypes and IgG subclasses in sera of 149 unselected RA patients. ACPA and anti-CarP antibody isotype and IgG subclass positivity, percentage and numbers (A). Levels of anti-CarP antibodies and ACPAs were plotted against each other, each isotype and IgG subclass separately (B–H). As internal control anti-CarP IgM and ACPA IgM were plotted against RF-IgM (I, J). Spearman Rank tests were performed to investigate correlations. HC; healthy controls, RA; rheumatoid arthritis, ACPA; anti-citrullinated protein antibodies, anti-CarP antibody; anti-carbamylated protein antibody, RF; rheumatoid factor, AU/ml; arbitrary units per millilitre. (TIF 55961 kb) [file 13075_2017_1392_MOESM4_ESM.tif]
