# Supplementary material for: The isotype and IgG subclass distribution of anti-carbamylated protein antibodies in rheumatoid arthritis patients
Source: Arthritis Res Ther. 2017 Aug 15;19:190. doi: 10.1186/s13075-017-1392-z (PMC5558706; doi:10.1186/s13075-017-1392-z)
Supplement: Supplementary file 5 — Associations of anti-CarP antibody isotypes and IgG subclasses with risk factors. Logistic regression univariate for risk factor analysis (smoking and SE positivity) IgG subclass analysis in anti-CarP IgG positive patients. Bold indicates a significant difference. * Remained significant after Holm-Bonferroni correction. Holm-Bonferroni correction separately for independent analyses in whole cohort. SE, shared epitope; OR, odds ratio; CI, confidence interval. (PDF 91 kb) [file 13075_2017_1392_MOESM5_ESM.pdf]

Associations of anti-CarP antibody isotypes and IgG subclasses with risk factors.

Isotypes	IgG- (n=190)	IgG + (n=184)	OR (95% CI)	p	IgA- (n=221)	IgA+ (n=152)	OR (95% CI)	p	IgM- (n=312)	IgM+ (n=61)	OR (95% CI)	p
**Smoking (ever) n (%)**	73 (41.5)	89 (51.7)	1.51 (0.99-2.31)	0.055	88 (42.9)	72 (50.7)	1.37 (0.89-2.10)	0.153	132 (45.5)	28 (49.1)	1.16 (0.66-2.04)	0.618
**SE positivity, n (%)**	108 (58.1)	137 (77.0)	2.41 (1.53-3.80)	**<0.001***	138 (64.5)	106 (71.1)	1.36 (0.86-2.13)	0.185	197 (64.8)	47 (79.7)	2.13 (1.08-4.18)	**0.029**

IgG subclasses	IgG1- (n=33)	IgG1+ (n=149)	OR (95% CI)	p	IgG2- (n=100)	IgG2+ (n=82)	OR (95% CI)	p	IgG3- (n=157)	IgG3+ (n=25)	OR (95% CI)	p	IgG4- (n=98)	IgG4+ (n=84)	OR (95% CI)	p
**Smoking (ever) n (%)**	14 (43.8)	73 (52.9)	1.44 (0.67-3.13)	0.352	48 (49.5)	39 (53.4)	1.17 (0.64-2.15)	0.611	80 (54.1)	7 (31.8)	0.40 (0.15-1.03)	0.057	43 (46.7)	44 (56.4)	1.48 (0.80-2.71)	0.209
**SE positivity, n (%)**	23 (71.9)	112 (77.8)	1.37 (0.58-3.25)	0.476	73 (74.5)	62 (79.5)	1.33 (0.65-2.71)	0.437	117 (76.5)	18 (78.3)	1.11 (0.38-3.19)	0.850	74 (77.1)	61 (76.3)	0.95 (0.47-1.92)	0.896

Logistic regression univariate for risk factor analysis (smoking and SE positivity)

IgG subclass analysis in anti-CarP IgG positive patients

Bold means significant difference, *remained significant after Holm-Bonferroni correction

Holm-bonferroni correction separately for independent analyses in whole cohort

SE, shared epitope; OR, odds ratio; CI, confidence interval
